# Neuromuscular Activation Patterns of the Gastrocnemius Muscle During Static and Dynamic Sprint Starts: Implications for Acceleration Training

**DOI:** 10.3390/jfmk11040387

**Published:** 2026-09-26

**Authors:** Ana Ferri-Caruana, Christian Castilla-Díaz, Angel Saez-Berlanga, Juan C. Colado

**Affiliations:** Research Group in Prevention and Health and Exercise and Sports (PHES), Department of Physical Education and Sports, University of Valencia, 46010 Valencia, Spain; ana.maria.ferri@uv.es (A.F.-C.); ccastilla330@gmail.com (C.C.-D.); angel.saez@uv.es (A.S.-B.)

**Keywords:** gastrocnemius, explosive acceleration, electromyography, biomechanics, injury prevention

## Abstract

**Objective**: The gastrocnemius muscle is crucial in explosive acceleration, yet the effect of sprint start type [static (began from an upright stance) vs. dynamic (included bilateral hops before sprinting)] on its activation remains underexplored. **Methods**: This study compared medial and lateral gastrocnemius activation during the first four contacts of 4 m sprints in 34 physically active males (24.13 ± 3.43 years) using surface electromyography normalized to peak sprint values. A two-way repeated-measures ANOVA (condition × foot contact) was used as the primary omnibus test (α < 0.05); given the significant interaction, simple-effects analyses—repeated-measures ANOVA within each start type and paired t-tests at each foot contact—were subsequently conducted, with the four pointwise start-type comparisons corrected for multiplicity using the Holm–Bonferroni method. **Results**: Overall gastrocnemius activation did not differ between conditions (static: 72.5% vs. dynamic: 71.7% of peak sprint EMG (*p* = 0.488, *d* = 0.12), and a significant condition × foot contact interaction (*p* = 0.001) confirmed that the temporal profile of activation across foot contacts differed between start types. Decomposing this interaction, the second foot contact showed the highest activation during static starts (significantly exceeding first and fourth, though not third foot contact), whereas during dynamic starts, the first contact was significantly lower than the second, third and fourth—which did not differ from one another. Between-condition comparisons at each foot contact showed that static starts exhibited a non-significant trend toward higher activation at the first and second foot contacts (*p* = 0.055 and *p* = 0.056, not significant after Holm–Bonferroni), while dynamic starts showed significantly greater activation at the fourth foot contact (*p* = 0.005, *d* = 0.52), the only pointwise comparison that survived correction for multiple comparisons (*p*_Holm = 0.02). **Conclusions**: These findings indicate an overall difference in the temporal pattern of GA activation between start types, with DS showing greater activation than SS at FC4, the only pointwise between-condition difference that survived multiplicity correction. Because activation did not differ among FC2, FC3, and FC4 within DS, the data do not demonstrate that the activation peak shifted specifically to FC4; furthermore, this EMG pattern does not demonstrate a reduction in muscle force, tendon strain, whole-limb mechanical loading, or injury risk, whereas the apparently higher early-phase activation in static starts at the first- and second-foot contacts should be interpreted as a non-significant trend, pending confirmation in larger samples. Sprint start type modulates gastrocnemius activation; whether these activation patterns translate into differences in performance or mechanical loading should be tested using concurrent kinetic, kinematic, and multi-muscle measurements.

## 1. Introduction

Explosive acceleration is essential in sports involving rapid force production, such as sprints, directional changes, and high-intensity actions [1]. It relies on neuromuscular efficiency and rapid muscular response. The gastrocnemius (GA) plays a key role by contributing to power output and stabilizing the ankle and knee joint [2].

Beyond the general demand for explosive force production, the initial acceleration phase of a sprint is characterized by specific kinematic determinants that dictate step-to-step performance. In world-class sprinters, trunk angle and thigh separation angle at take-off from the first ground contact after block exit explain nearly 90% of the variance in normalized horizontal external power, underscoring the postural and inter-segmental coordination required to generate an effective early impulse [3]. Ankle joint mechanics also play a central role during this window: a greater range of ankle dorsiflexion during early stance is significantly associated with a higher ratio of horizontal-to-resultant forces during the initial acceleration steps [4]. Because the GA spans the ankle and contributes directly to the plantarflexion moment generated immediately after this dorsiflexion phase, these kinematic findings provide a direct biomechanical rationale for examining its activation patterns specifically within the first foot contacts of the sprint, rather than across the acceleration phase as a whole.

Athletes use strategies such as the False Step (brief backward motion) or the Forward Step (direct propulsion). Although the False Step may increase knee joint loading [5], its prevalence suggests a neuromechanical trade-off that enhances horizontal force production [6]. These techniques, including the static start (SS) and dynamic start (DS), aim to optimize the GA’s role in power generation and stabilization. In SS, the GA responds to high mechanical demands during initial contact, including stretch–shortening cycles and center-of-mass adjustments [7]. DS, initiated with bilateral hops, may exploit pre-activation and elastic energy to reduce these demands, despite increased motor control requirements.

The physiological rationale for expecting reduced early-phase demand in DS is grounded in the stretch–shortening behavior of the ankle plantarflexion muscle–tendon unit during the push-off action. High-speed video analyses of the sprint start have shown that the soleus muscle–tendon unit undergoes a marked dorsiflexion of the ankle immediately before plantarflexion during block clearance, a sequence consistent with a stretch–shortening-cycle-like mechanism that appears to enhance force generation during push-off across sprinters of varying performance levels [8]. Because the bilateral hops preceding the dynamic start are expect to pre-tension the triceps surae complex in a comparable manner, this mechanism offers a plausible physiological explanation for why peak GA activation may be delayed relative to the SS, shifting from the earliest contracts toward later, more elasticity-dependent phases of the acceleration.

Electromyographic (EMG) analyses show early, high-amplitude GA activation during initial acceleration [9], underscoring its key role in propulsion. The medial GA is frequently injured in explosive sports, likely due to its larger cross-sectional area and primary involvement during push-off phases [10,11]. These injuries typically occur during ballistic actions involving ankle dorsiflexion and knee extension, such as standing accelerations or rapid direction changes.

Although the present study focuses on linear sprint acceleration, comparable start technique decisions are ubiquitous in ball game and team sports, where athletes must translate a stationary or quasi-stationary stance into rapid, direction-specific acceleration. In lateral-direction, short-distance sprints typical of ball sports, the forward-step technique has been shown to outperform the false-step technique in both average and terminal sprint velocity, an advantage attributed to greater acceleration achieved in the initial phase immediately after movement initiation [12]. This body of evidence indicates that the trade-off between mechanically preparatory movements—conceptually analogous to the bilateral hops of the DS investigated here—and immediate forward propulsion—analogous to the SS—is not confined to track sprinting, but is directly relevant to invasion and court sports such as basketball, where explosive first-step acceleration from a defensive or transitional stance is a recurrent performance determinant.

Despite its relevance, the effect of start types on early-phase GA activation remains underexplored. This study compared GA activation during the first four-foot contacts (FC1–FC4) in SS and DS. Because acceleration mechanics are known to evolve non-uniformly across consecutive foot contacts, we also tested whether the effect of start type on GA activation varied across FC1–FC4, i.e., a condition × foot contact interaction. We hypothesized that SS would elicit greater activation at FC1 due to higher stabilization demands, and that FC1 would show the highest overall activation in both conditions.

## 2. Methods

### 2.1. Participants

Thirty-four physically active males (age = 24.13 ± 3.43 years; 178.97 ± 6.53 cm; 72.44 ± 9.08 kg) participated. An a priori power analysis (G*Power v3.1; Heinrich Heine University Düsseldorf; Düsseldorf, Germany) indicated that 32 participants were required to detect a large effect (f = 0.40; α = 0.05; power = 0.80).

Inclusion criteria were: (i) aged 18–40 years, (ii) regular physical activity (≥30 min/day, ≥3 times/week), and (iii) no musculoskeletal injuries in the past six months. Exclusion criteria included GA rupture, lower limb or lumbar injuries, persistent pain, neurological or vestibular disorders, or contraindications to physical exertion. All participants engaged in a minimum of 3 h per week of structured physical activity, but this activity differed in nature across the sample. A subset of participants was physically active in disciplines involving resistance training or intermittent team-sport activity (e.g., football, handball, or basketball), whereas the remainder were sprint- or athletics-trained individuals whose training was limited to running-based conditioning. This distinction is relevant because habitual sprint-specific training may influence neuromuscular coordination and start-technique proficiency and should therefore be considered when generalizing the present activation patterns to populations with different training backgrounds. Participants avoided strength or running training for 48 h before testing. Leg dominance was determined by the preferred kicking leg [13].

All participants were fully informed about this study and signed written consent. The protocol was approved by the Human Research Ethics Committee of the University of Valencia (Ref. 1973315) and complied with the Declaration of Helsinki.

### 2.2. Experimental Procedure

Testing was conducted in a single day on an outdoor synthetic track. Participants performed a standardized warm-up with aerobic and dynamic stretching, followed by submaximal sprints and familiarization with static and dynamic start protocols, as previously described.

Participants completed six 4 m maximal accelerations (three SS, three DS). The fastest trial per condition, identified via Kinovea (v.0.9.4; Kinovea Open Spurce Project; Burdeos, France), was used for analysis. This selection was intended to characterize GA activation associated with each participant’s best 4 m performance in each condition; accordingly, the resulting estimates should be interpreted as best-performance rather than average-trial responses. To minimize order effects, the sequence in which the two start conditions were performed was determined by simple randomization. A coin toss was conducted for each participant prior to testing, with heads assigned a priori to the DS and tails to the SS, thereby determining which condition was performed first. The full six-trial sequence followed a strictly alternating pattern rather than being grouped by condition. Once the starting condition had been determined by the coin toss, subsequent trials alternated between the two start types throughout the session: a participant randomized to begin with the DS completed the sequence (1) DS, (2) SS, (3) DS, (4) SS, (5) DS, (6) SS, with the reverse order applied to participants who began with the SS. A standardized passive recovery period of 3 min was enforced between all six maximal-effort attempts. This interval was selected to allow near-complete resynthesis of intramuscular phosphocreatine stores, since full restoration of phosphocreatine following high-intensity muscular effort has been reported to require up to approximately 3 min in human skeletal muscle [14], thereby limiting the influence of cumulative neuromuscular fatigue on the activation patterns compared across alternating SS and DS trials.

Before their first attempt at each start type, participants received a visual demonstration of the corresponding technique and then completed one execution of that start, which served as a condition-specific familiarization trial to ensure adequate understanding of the movement pattern before data collection began for that condition. SS began from an upright stance; DS included bilateral hops before sprinting. A verbal cue ensured synchronized timing. Two 40 m sprints were used to determine peak EMG values for normalization (see Figure 1 for more details).

### 2.3. Electromyographic Measurements

EMG activity of the medial and lateral gastrocnemius (MG, LG) was recorded bilaterally using two mDurance units (1024 Hz), with a separate channel for each muscle head. For the present analysis, only the MG and LG signals from the dominant leg were retained in order to characterize the activation profile across both gastrocnemius heads rather than a single head in isolation. The mDurance system (mDurance Solutions SL, Granada, Spain) used in this study is a portable two-channel surface EMG platform built around the Shimmer3 EMG unit (Realtime Technologies Ltd., Dublin, Ireland), sampling at 1024 Hz with 24-bit resolution and an amplification range of 100–10.000 V/V. Its concurrent validity against a laboratory-grade reference system (Delsys Trigno) has been established during dynamic isokinetic contractions, showing almost perfect to substantial intraclass correlation coefficients (ICC > 0.81) [15]. Although this validation was conducted on knee extensor musculature rather than the triceps surae, it supports the general concurrent validity of the device’s hardware and signal-processing pipeline for dynamic, non-isometric tasks comparable in nature to the present sprint-start protocol. Electrodes (10 mm inter-electrode distance) were placed according to SENIAM guidelines after shaving and cleaning the skin [16]. A reference electrode was positioned over the lateral malleolus. Signals were processed using a fourth-order Butterworth bandpass filter (20–450 Hz) to remove motion artifacts. Signals <0.05 V or >2 V were excluded via automated detection and visual inspection.

EMG data were normalized to the mean of the two highest peaks from 40 m sprints, following dynamic normalization protocols [17], and expressed as a percentage of peak sprint EMG. The choice of a dynamic, task-specific normalization procedure over a classic maximum voluntary isometric contraction (MVIC) was deliberate. Methodological comparisons of amplitude-normalization approaches have shown that the reference contraction selected shapes both the magnitude and the between-participant variability of the resulting EMG pattern, to the point of altering the physiological interpretation of the signal; MVIC-based normalization tends to introduce greater inter-individual variability, while task-based peak normalization reduces variability at the cost of discarding absolute amplitude information [18]. Given that the sprint start is an explosive, multi-joint, dynamic action markedly different in muscle length, contraction velocity, and coordination pattern from a static isometric contraction, normalizing GA EMG to the mean of the two highest RMS peaks recorded during maximal 40 m sprints was considered a more ecologically valid reference than an isolated MVIC, since it preserves the specific contractile conditions (velocity, joint angle, and stretch–shortening demand) under which the muscle is required to act during the task of interest.

Foot-ground contacts were identified through a synchronized video-EMG recording function embedded in the mDurance application, which simultaneously captures a video stream of the trial via a smartphone camera (60 frames per second; ≈16.70 ms) alongside the real-time neuromuscular activation trace of the selected muscles. This concurrent display allowed each foot contact to be visually cross-referenced against its corresponding EMG burst within the same recording session, rather than requiring post hoc alignment of two independently acquired signals. The video stream was reviewed frame-by-frame to mark the instant of initial ground contact for FC1 to FC4, which was then matched to the nearest EMG sample for peak RMS extraction. The main variable was peak RMS EMG at foot contacts FC1-FC4. Although MG and LG activity was recorded bilaterally, only the dominant-leg signals were used in the statistical analysis. For each foot contact, the peak RMS values of the two heads were averaged into a single composite GA activation value, which constituted the dependent variable entered into the statistical analysis. MG and LG activation were therefore not analyzed as separate factors (see Figure 2 for more details).

Foot-contact-related muscle bursts were further characterized, in addition to visual verification against the synchronized video, using amplitude- and time-based activation thresholds. Onset and offset of each contraction were defined using (i) two consecutive 50 ms time windows (100 ms total), establishing the minimum period required to confirm that the muscle had returned to a relaxed state, and (ii) an amplitude threshold of 18% of peak muscular activity, which set the intensity level required to mark the start and end of a contraction [19]. Because ballistic, high-amplitude contractions such as those occurring during sprint starts produce EMG bursts with steeper rise times and less baseline overlap than those typical of postural or submaximal tasks, the conventional 5% threshold commonly used in EMG onset-detection literature was considered insufficiently conservative for this protocol [20]; the 18% threshold was therefore adopted to reduce the risk of false-positive onset detection from low-amplitude baseline noise.

### 2.4. Statistical Analysis

Statistical analyses were performed in SPSS v26 (IBM Corp., Armonk, NY, USA). Data are presented as mean ± standard deviation (SD). A two-way repeated-measures ANOVA (condition × foot contact) was performed as the primary omnibus test, with Greenhouse–Geisser correction applied when sphericity was violated. Given the significant condition × foot contact interaction, simple-effects analyses were subsequently conducted: (i) a repeated-measures ANOVA, with Greenhouse–Geisser correction when sphericity was violated and Bonferroni-adjusted post hoc tests, examined the effect of foot contact within each start type; and (ii) paired t-tests examined the effect of start type at each foot contact, with the four resulting *p*-values corrected for multiplicity using the Holm–Bonferroni method (α = 0.05) [21]. Effect sizes were reported as partial eta squared (η^2^p) classified as small (0.01), moderate (0.06), and large (0.14), following standard conventions for repeated-measures designs [22]. Given the exploratory nature of some pairwise contrasts, effect sizes are reported alongside 95% confidence intervals rather than relying on significance thresholds alone. Significance was set at *p* < 0.05.

## 3. Results

### 3.1. Overall GA Activation: Static vs. Dynamic Start

GA activation (%peak sprint EMG) did not differ significantly between start types: 72.50 ± 9.60% in SS vs. 71.70 ± 11.00% in DS (t(33) = 0.70, *p* = 0.49, *d* = 0.12). Both values were ~30% lower than activation during the maximal 40 m sprint.

### 3.2. Intra-Condition Analysis of Foot Contacts in Static and Dynamic Starts

In SS, repeated-measures ANOVA revealed a significant effect of foot contact on GA activation: F(3, 99) = 8.25, *p* < 0.001, η^2^p = 0.20, with sphericity met (Mauchly’s W = 0.76, *p* = 0.12). Bonferroni-adjusted post hoc tests showed that FC2 had significantly higher activation than FC1 (*p* < 0.001), FC3 (*p* = 0.13), and FC4 (*p* = 0.03). FC1 was also significantly lower than FC3 (*p* = 0.01); FC1 vs. FC4 and FC3 vs. FC4 remained non-significant (both *p* = 1.00).

In DS, ANOVA showed a significant effect of foot contact: F(2.45, 80.95) = 15.20, *p* < 0.001, η^2^p = 0.23, with sphericity violated (Mauchly’s W = 0.64, *p* = 0.02); the Greenhouse–Geisser correction was applied (ε = 0.82). Post hoc analysis revealed that FC1 activation was significantly lower than FC2, FC3, and FC4 (*p* < 0.001 for all three contrasts), with no differences among FC2-FC4 (*p* > 0.05).

### 3.3. Two-Way ANOVA: Condition (Foot Contact Interaction)

A two-way repeated-measures ANOVA (condition × foot contact) was performed as the primary omnibus test. There was no significant main effect of condition (F(1,33) = 0.49, *p* = 0.49, η^2^p = 0.02), consistent with the overall comparison in Section 3.1. The main effect of foot contact was significant (F(3,99) = 14.31, *p* < 0.001, Greenhouse–Geisser-corrected *p* < 0.001, η^2^p = 0.30). Critically, the condition × foot contact interaction was significant (F(3,99) = 5.69, *p* = 0.001, Greenhouse–Geisser-corrected *p* = 0.002, η^2^p = 0.15), confirming that the temporal profile of GA activation across foot contacts differs between static and dynamic starts (see Figure 3 and Table 1 for descriptive values and pointwise contacts). This interaction is the statistical basis for the pointwise contrasts reported in the following section, and should be regarded as the primary between-condition finding of this study.

### 3.4. Between-Condition Comparison at Each Foot Contact

Paired *t*-tests revealed differing GA activation patterns between SS and DS at specific foot contacts: at FC1 (66.10 ± 12.30% vs. 60.20 ± 16.30%, t(33) = 1.99, *p* = 0.055, *d* = 0.34, not significant after Holm–Bonferroni correction, *p*_Holm = 0.164) and FC2 (80.70 ± 16.80% vs. 76.20 ± 17.20%, t(33) = 1.98, *p* = 0.056, *d* = 0.34, not significant after correction, *p*_Holm = 0.164), SS showed numerically higher mean activation; however, neither comparison was statistically significant before or after correction for multiple comparisons. At FC4, DS was significantly higher (76.50 ± 13.40% vs. 69.40 ± 16.80%, t(33) = –3.01, *p* = 0.005, *d* = –0.52, the only comparison surviving Holm–Bonferroni correction, *p*_Holm = 0.020). No differences were found at FC3 (73.80 ± 11.90% vs. 73.80 ± 12.00%, t(33) = 0.03, *p* = 0.98, *d* = 0.00) (see Table 1 and Figure 4).

Distributional checks on the SS-DS paired differences (Shapiro–Wilk) confirmed normality at FC1, FC2, and FC4 but detected a deviation from normality at FC3 (W = 0.87, *p* < 0.001). A Wilcoxon signed-rank sensitivity analysis on the four contacts reproduced the same pattern of statistical decisions as the paired t-tests (FC1 and FC2 marginal/non-significant, FC3 non-significant, and FC4 significant).

## 4. Discussion

This study identified a significant condition × foot contact interaction, indicating that the temporal profile of GA activation differed between start types. SS showed numerically greater mean activation at FC1 and FC2, but neither between-condition contrast was statistically significant before or after Holm–Bonferroni correction. DS showed significantly greater activation than SS at FC4; however, because activation did not differ among FC2, FC3, and FC4 within DS, FC4 should not be described as a shifted activation peak. These findings have practical relevance for optimizing performance and, given that acceleration has been identified as the most common inciting mechanism for GA strain injury in explosive sports [23], may also carry implications for injury risk—although this latter interpretation remains hypothesis-generating rather than confirmatory in explosive sports.

Although overall GA activation did not differ between SS and DS, activation patterns across foot contacts were distinct, as confirmed by a significant condition × foot contact interaction (*p* = 0.001). SS showed a non-significant trend toward greater activation at FC1-FC2 (*p* = 0.055, and *p* = 0.056, respectively; neither was significant after correction for multiple comparisons), which may reflect the absence of pre-activation and the need to generate force from rest, consistent with kinetic data showing greater joint moments and inertial demands in early SS steps [4], and with kinematic evidence that postural and inter-segmental adjustments at these very first contacts are a major determinant of effective early impulse generation [3,4]. In contrast, DS significantly shifted (*p* = 0.005, surviving multiplicity correction) activation toward FC4, likely due to a pre-activation through a stretch–shortening cycle mechanism specific to the sprint-start push-off: high-speed analyses have shown that the ankle plantar flexor muscle–tendon unit undergoes a marked stretch immediately before shortening during block/hop clearance, a sequence associated with enhanced force generation [8], which may influence energy storage and the timing of GA activation; however, the present EMG data cannot establish a reduction in early concentric load [24]. This finding also runs contrary to our initial hypothesis that FC1 would show the highest activation irrespective of start type: instead, FC1 was consistently the point of lowest activation in DS, suggesting that pre-activation from the preceding hops, rather than postural stabilization demands, is the dominant determinant of the early-phase activation profile in this condition.

The greater FC2 activation (vs. FC1) contradicts our hypothesis but aligns with biomechanical interpretations. At FC2, the center of mass is more forward, and hip/knee extension improves mechanical leverage for propulsion [25]. Comparatively lower FC1 activation likely reflects shorter contact time and limited joint flexion immediately after start initiation, despite its key role in initiating acceleration [26,27]; this is consistent with kinematic reports that the postural and inter-segmental configuration needed for effective horizontal force transmission is only fully achieved from the second contact onward [3].

The numerical FC1-FC2 pattern, together with the confirmed lower FC1 activation in DS, is consistent with a hypothesis that DS may shift peak GA electrical activation to a later foot contact relative to SS. Although MG is disproportionately represented among GA strain injuries [11,23,28], the present composite outcome cannot determine whether the observed condition × foot contact pattern was driven by MG, LG, or both; therefore, head-specific injury implications remain speculative. This activation pattern warrants confirmation in a larger, adequately powered sample before being used to guide injury-prevention recommendations. However, whether this activation pattern reflects reduced mechanical loading, a protective effect, or a trade-off with propulsion remains to be established in short (<5 m) accelerations. These findings align with the common use of the “false step” in team sports, where athletes must convert a stationary or transitional stance into rapid, direction-specific acceleration in a manner conceptually analogous to the SS-versus-DS comparison examined here [12]. Despite increasing knee loads [29], this strategy enhances force production by repositioning the center of mass and activating the stretch–shortening cycle [6]. DS mimics this via pre-activation hops, promoting elastic-driven force and shifting muscular demand.

The use of dynamic normalization based on peak EMG during 40 m sprints improved ecological validity [17], consistent with methodological evidence that the reference contraction selected shapes both the magnitude and inter-individual variability of the resulting EMG pattern, favoring task-specific over isometric references for dynamic, ballistic actions such as the sprint start [18]. While force plates were not used, consistency between EMG patterns (e.g., FC1–FC2) and published kinetic data [30] supports the validity of this field-based approach, further supported by prior concurrent validity testing of the mDurance system against a laboratory-grade reference system during dynamic contractions [15].

Practically, these findings support differentiated training strategies. SS may be useful when the aim is to emphasize early GA activation at sprint initiation, a quality directly relevant to court and invasion sports such as basketball, where explosive first-step acceleration from a defensive transitional stance is a recurrent performance determinant, and where individualized acceleration-oriented training has been shown to improve multidirectional speed within a few weeks [31]. Conversely, DS may be useful when the aim is to emphasize later GA activation; its effects on elastic efficiency, mechanical loading, injury prevention, and rehabilitation outcomes remain to be established, consistent with evidence that both unilateral and bilateral explosive training modalities can be selectively targeted to different components of sprint and change-of-direction performance in basketball players [32]. Based on GA activation patterns, we tentatively propose, as a theoretically derived framework requiring empirical validation, a periodization model: (i) preparatory: 3:1 SS:DS to emphasize earlier GA activation; (ii) competitive: 1:1 to provide exposure to both activation patterns; (iii) rehabilitation: 1:3 to emphasize later GA activation as an exploratory option, without assuming reduced tissue loading. This activation-based framework requires validation with kinetic and prospective injury outcomes before it can inform load-management, rehabilitation, or injury-prevention prescriptions.

This study has some limitations: (i) a further limitation concerns measurement reliability. Because only the fastest trial of each condition, identified through video-based timing, was retained for analysis, the present design does not allow the trial-to-trial reproducibility of GA activation to be established. Future studies should retain all recorded attempts within each condition and compute a two-way random-effects, absolute-agreement ICC (A,1), following the reporting guidelines of Koo and Li [33], before the peak activation values reported here are used as normative benchmarks for training monitoring or return-to-sport decision-making. Accordingly, the present findings describe the neuromuscular activation pattern of the selected fastest performance and should not be interpreted as estimates of the average response across attempts or of within-session repeatability. An analysis retaining all three trials in a linear mixed-effects model, with participant included as a random effect and condition, foot contact, within-condition repetition, and their relevant interactions included as fixed effects, would estimate the average condition effect while accounting for within-participant dependence and would test whether the reported condition × foot contact interaction is robust across attempts. Analyses of each repetition separately could complement this model as sensitivity analyses of consistency, but would be statistically less efficient and would require control for multiple testing and possible order effects. Because these alternative analyses were not performed, their direction and magnitude cannot be inferred from the present results; (ii) the was conducted under a closed, self-initiated protocol in which both start conditions were triggered by a sample, standardized verbal cue requiring a single pre-determined motor response, rather than under the choice-reaction and perceptual-decision demands typical of competitive match play, such as reacting to an opponent’s movement or selecting a start direction under time pressure. This distinction is not merely contextual but physiologically meaningful: recent evidence in female football players has shown that the presence versus absence of anticipatory information fundamentally alters lower-limb neuromuscular control, with unanticipated movement execution producing longer ground contact times and less coordinated trunk–limb activation patterns than anticipated execution of the same biomechanical task [34]. Because the GA activation profiles reported here were obtained under fully anticipated, cued conditions, they may not generalize to the reactive, decision-laden starts required in invasion and court sports, where athletes must select and execute a start technique in direct response to an opponent or a game-related stimulus. Future research should therefore examine GA activation during reactive, sport-specific start tasks incorporating an unpredictable external trigger, such as a visual or auditory go-signal requiring a directional choice, to determine whether the condition × foot contact interaction identified here is preserved under such conditions or is instead partly attributable to the closed, anticipated nature of the present task.

(iii) Fatigue was not experimentally induced or evaluated in this protocol, despite its well-documented influence on motor control and lower-limb injury risk. In a simulated football match, hamstring fatigue has been shown to reduce maximal isometric torque and to be associated with kinematic alterations during running that could plausibly elevate injury risk [35]. Although that evidence pertains to the hamstrings rather than the GA, the underlying mechanism, whereby accumulated fatigue alters neuromuscular output and subsequently shifts joint kinematics, is directly transferable as a working hypothesis to the triceps surae during repeated sprint starts. Future protocols incorporating a standardized fatiguing task prior to data collection would help clarify whether the condition × foot contact interaction reported here is preserved, attenuated, or exacerbated under fatigued conditions, with direct implications for load management strategies in high-volume training or competition periods. (iv) The pointwise differences observed at FC1 and FC2, with SS showing numerically greater activation than DS, did not survive correction for multiple comparisons in the present male-only sample of 34 participants (*p*_Holm = 0.164 for both contacts). Given that the associated effect sizes were of small-to-moderate magnitude, it remains plausible that these early-phase differences are genuine but under-powered in the current dataset rather than absent. Future studies with larger and sex-inclusive samples are needed to determine whether the apparent early-phase advantage of SS represents a reproducible pattern or a sampling artifact, and whether it generalizes beyond the male, physically active population studied here.

(v) While the present findings and the proposed SS/DS periodization model are framed for physically active and athletic populations, it is worth considering whether comparable GA activation patterns are relevant beyond sport-specific contexts. Future research could explore whether similar activation strategies are meaningful for the reactive neuromuscular control of the ankle plantar flexors in older adults, for example during balance recovery following a trip or slip. However, this extension should be approached with caution: prior work examining knee and ankle extensor muscle–tendon properties in relation to balance recovery from a simulated slip in older adults found no clear association between these properties and recovery success, and concluded that explosive strength training in isolation is unlikely to be sufficient for fall prevention [36]. Any future application of the present activation paradigm to non-athletic or aging populations should therefore be considered exploratory and should not be assumed to translate directly into fall prevention efficacy. (vi) It should be noted that surface EMG amplitude, even after peak-based normalization, provides an index of the muscle’s electrical activation rather than a direct measure of the mechanical force, tendon strain, or joint moment it produces. The relationship between EMG amplitude and force outputs is shaped by motor unit recruitment strategy, electromechanical delay, and several non-physiological recording factors, and can become non-linear under fatigue or during rapid, ballistic contractions such as those examined in the present sprint-start protocol [37]. Consequently, the present activation-based interpretation of GA involvement across foot contacts cannot, by itself, establish the actual magnitude of mechanical loading experienced by the muscle–tendon unit during each contact. Accordingly, a lower normalized EMG amplitude should not be interpreted as lower mechanical loading. Combining surface EMG with synchronous ground reaction force measurement and three-dimensional motion capture would allow joint kinetics, muscle-tendon strain, and neuromuscular activation to be examined concurrently, offering a substantially more complete picture of musculoskeletal loading during the acceleration phase and providing a stronger empirical basis for any future inferences regarding injury risk.

(vii) Foot contacts were identified from synchronized 60 fps video, which imposes a temporal uncertainty of up to one frame (approximately 16.70 ms). Although this resolution was adequate for assigning FC1–FC4 to their corresponding EMG bursts and extracting peak RMS amplitude, higher-speed video, force plates, or instrumented contact detection would be required for analyses focused on fine-scale onset timing or electromechanical delay. (viii) Finally, a further limitation concerns the level of muscular and limb specificity captured by the present design. Although EMG activity was recorded bilaterally, the statistical analysis was restricted to the dominant leg, and the MG and LG heads were averaged into a single composite activation value rather than analyzed separately. No EMG data were obtained from other muscles involved in sprinting (e.g., the soleus, tibialis anterior, quadriceps, hamstrings, or gluteus maximus); therefore, this study cannot determine whether neuromuscular demand was redistributed to unmeasured muscles. This approach cannot detect head-specific or between-limb differences in the condition × foot contact pattern reported here. This is a non-trivial simplification given that the MG is disproportionately represented among GA strain injuries relative to the LG [23], and that between-limb asymmetries are themselves a recognized risk factor in explosive sports. Future studies should analyze both limbs and the MG and LG as separate factors to determine whether the interaction identified here is uniform across heads and limbs or concentrated in one of them.

## 5. Conclusions

Sprint start type did not change overall GA activation but did alter its temporal pattern across the first four contacts, as indicated by the significant condition × foot contact interaction. The most robust pointwise finding was greater GA activation with DS than SS at FC4; because activation did not differ among FC2, FC3, and FC4 within DSA, this result should not be interpreted as a shift of the activation peak specifically to the fourth contact. The most robust, statistically confirmed finding is that DS shifts peak GA activation to the fourth contact relative to SS, indicating a later peak in GA electrical activation; whether this pattern corresponds to reduced early-phase mechanical loading requires confirmation with kinetic measurements. A trend toward higher SS activation at the first and second foot contacts was also observed but did not reach significance and should be treated as hypothesis-generating rather than confirmatory. These findings may inform acceleration programs intended to vary the timing of GA activation, including in team and court sports such as basketball, where explosive first-step acceleration from a stationary or transitional stance is a recurrent performance determinant; the SS:DS periodization frameworks outlined in this study should, however, be regarded as theoretically derived, activation-based hypotheses requiring empirical validation with mechanical loading and prospective injury outcomes rather than as validated training, rehabilitation, or injury prevention prescriptions, while underscoring the need for adequately powered replication of the early-phase (FC1-FC2) contrasts, together with future studies incorporating sex-inclusive samples, fatigue and reactive, sport-specific start protocols, and formal test–retest reliability analyses of the activation measures reported here.

## Figures and Tables

**Figure 1:**
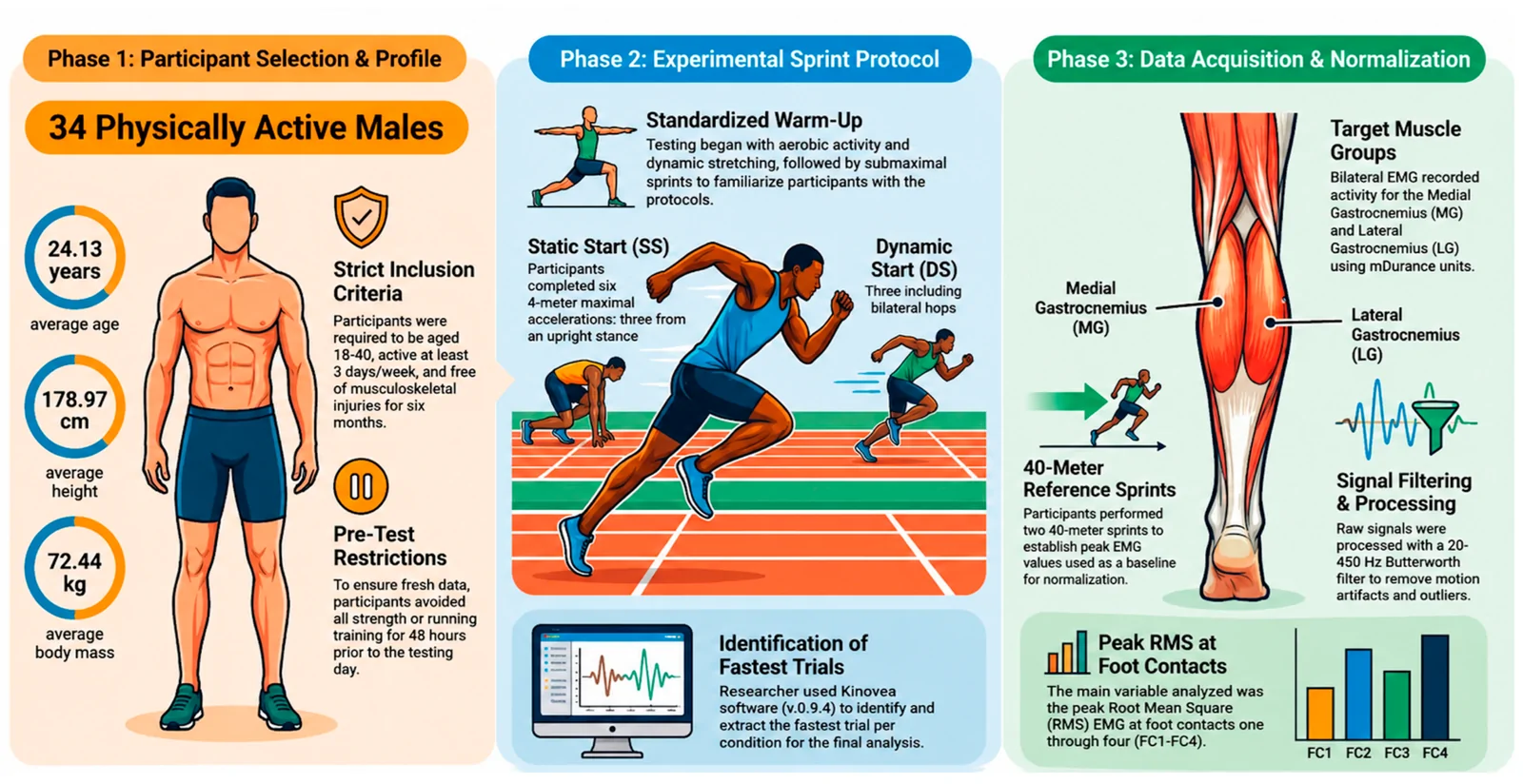
Study protocol. SS, static start; DS, dynamic start; MG, medial gastrocnemius; LG, lateral gastrocnemius; EMG, electromyography; RMS, root mean square; FC, foot contact.

**Figure 2:**
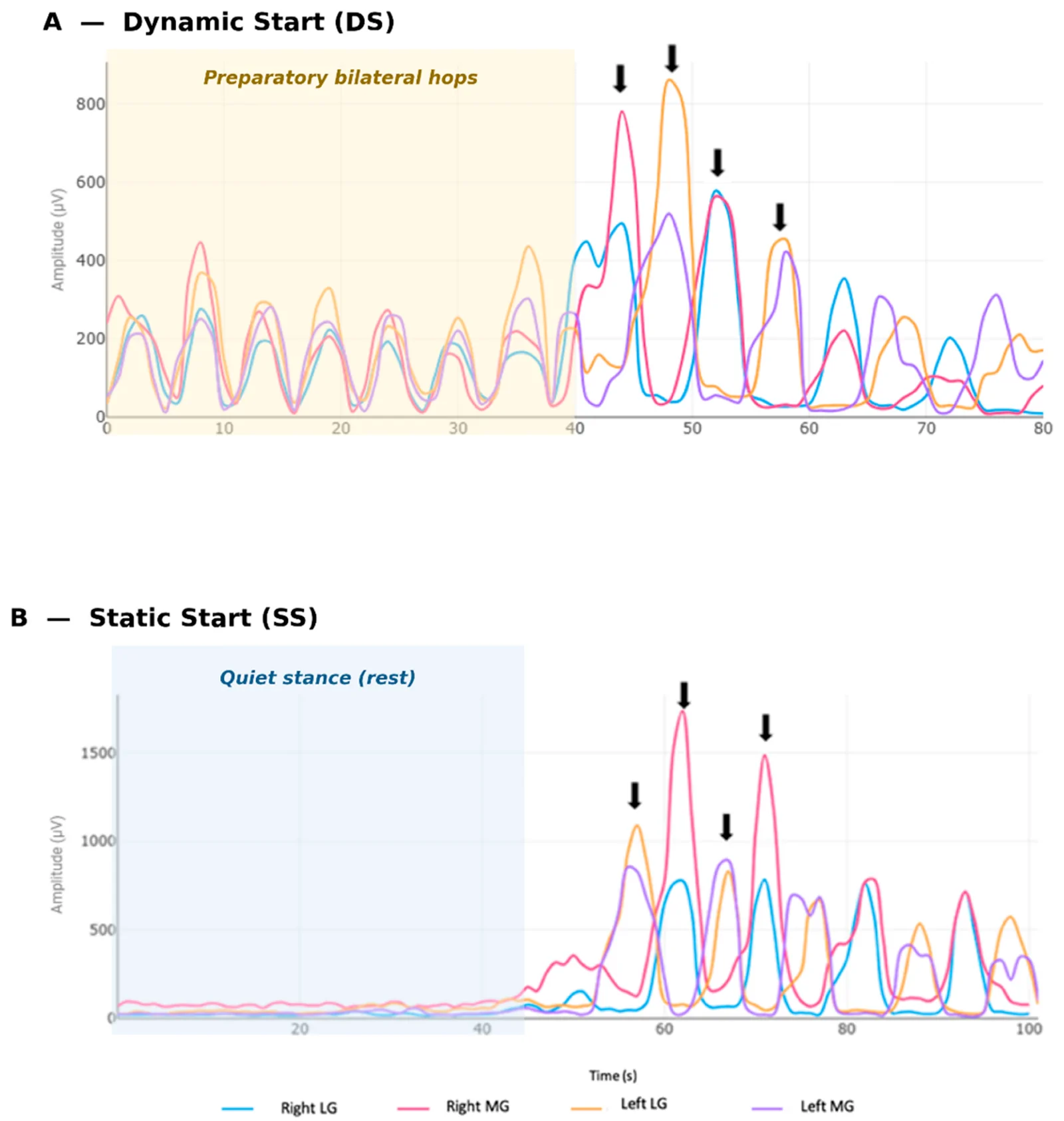
Representative gastrocnemius EMG traces during dynamic (**A**) and static (**B**) sprint starts. DS, dynamic start; SS, static start; LG, lateral gastrocnemius; MG, medial gastrocnemius. Note. The four traces show bilateral MG and LG signals from a single representative participant per condition (not group-averaged data); only the dominant-leg signals were used in the inferential analysis. Black arrows indicate the four analyzed foot contacts (FC1–FC4).

**Figure 3:**
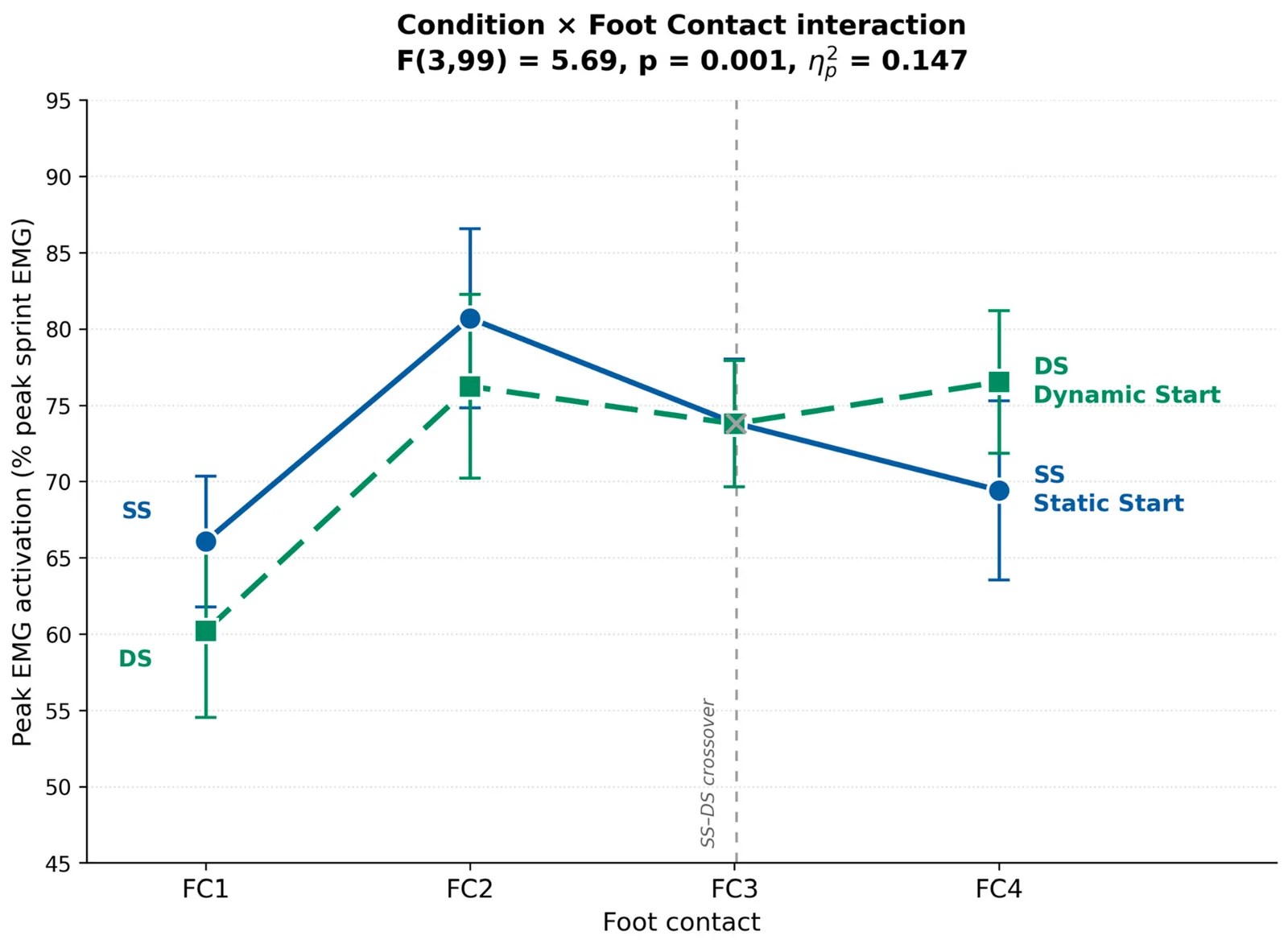
Condition × foot contact interaction in gastrocnemius peak EMG activation (*N* = 34). The static-start series (SS; blue, solid line, circular markers) shows numerically higher mean activation at the first and second foot contacts, the two conditions converge at the third, and the dynamic-start series (DS; green, dashed line, square markers) shows higher mean activation at the fourth. Series are labeled directly on the plot. Error bars represent the 95% confidence interval of the mean; the vertical guide line marks the SS–DS crossover between the third and fourth foot contacts underlying the significant interaction. FC, foot contact. Data expressed as % peak sprint EMG.

**Figure 4:**
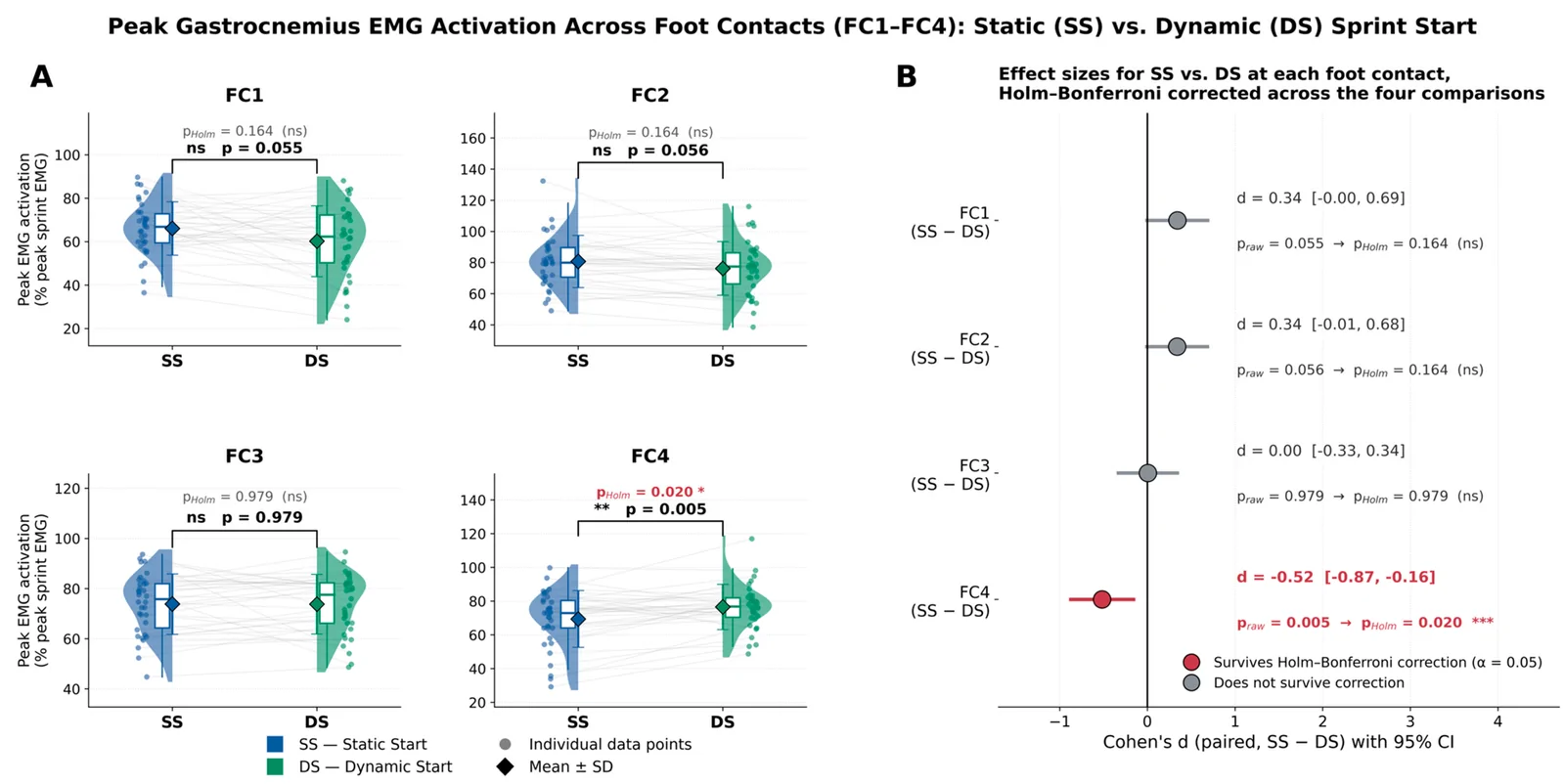
Peak gastrocnemius EMG activation across foot contacts and effect-size summary for static (SS) versus dynamic (DS) sprint starts (*N* = 34). (**A**) Raw-data distributions at each foot contact (FC1–FC4); half-violin plots show the kernel density estimate, boxes show the interquartile range and median, dots show individual participants, gray lines connect paired observations, and diamonds show mean ± SD. Asterisks denote the raw paired *t*-test result at each contact; *p*_Holm values indicate significance after Holm–Bonferroni correction applied jointly across the four comparisons. (**B**) Forest plot of the paired effect sizes (Cohen’s *d)* for the same four comparisons, with 95% confidence intervals; red markers indicate comparisons that remain significant after Holm–Bonferroni correction (α = 0.005), gray markers indicate comparisons that do not. Data expressed as % peak sprint EMG. * *p* < 0.05, ** *p* < 0.01, *** *p* < 0.001 (raw, per-comparison), ns: no significant.

**Table 1:** Between-condition (SS vs. DS) comparison of gastrocnemius peak EMG activation at each foot contact.

Foot Contact	SS Mean ± SD	DS Mean ± SD	t	*p*_Raw	Cohen’s *d* [95% CI]	*p*_Holm
FC1	66.10 ± 12.30	60.20 ± 16.30	1.99	0.055	0.34 [0.00,0.69]	0.164
FC2	80.70 ± 16.80	76.20 ± 17.20	1.98	0.056	0.34 [–0.001,0.68]	0.164
FC3	73.80 ± 12.00	73.80 ± 11.90	0.03	0.979	0.00 [–0.33,0.34]	0.979
FC4	69.40 ± 16.80	76.50 ± 13.40	–3.01	0.005	–0.52 [–0.87, –0.16]	0.020

Note. SS, static starts; DS, dynamic starts; SD, standard deviation; CI, confidence interval; FC, foot contact.

## Data Availability

Data used in this study are part of a larger ongoing project. Access to the data can be provided upon reasonable request to the corresponding author.
